# Comparative Hepatoprotective Activity of Ethanolic Extracts of *Cuscuta australis* against Acetaminophen Intoxication in Wistar Rats

**DOI:** 10.1155/2014/730516

**Published:** 2014-09-10

**Authors:** Rachael O. Folarin, Jamiu O. Omirinde, Ronald Bejide, Tajudeen O. Isola, Levi I. Usende, Afisu Basiru

**Affiliations:** ^1^College of Health Sciences, Ladoke Akintola University of Technology, Ogbomoso 4000, Nigeria; ^2^Department of Veterinary Anatomy, University of Jos, Jos 930001, Nigeria; ^3^Department of Morbidity and Forensic Medicine, Obafemi Awolowo University, Ile Ife A234, Nigeria; ^4^Department of Veterinary Public Health, Anatomy & Physiology and Pharmacology, Ibadan 900001, Nigeria

## Abstract

This study investigates the comparative hepatoprotective activity of crude ethanol extracts of* Cuscuta australis* against acetaminophen (APAP) intoxication. Thirty-six rats were randomly divided into six groups of 6 replicates: Group 1 which served as control received water. Group 2 was orally administered 835 mg/kg body wt. of paracetamol on day 8. Groups 3 and 4 were orally administered ethanolic extracts of the seed of* Cuscuta australis* in doses of 125 mg/kg and 250 mg/kg, respectively, for 7 days and then intoxicated as in Group 2 on the 8th day. Groups 5 and 6 received similar oral doses of* Cuscuta australis* stem extracts for 7 days and then intoxicated as in Groups 3 and 4. Group 2 rats showed severe periportal hepatic necrosis, significantly elevated serum hepatic injury markers, markedly increased lipid peroxidation, and decreased hepatic antioxidant enzymes activities. Remarkably,* Cuscuta australis* (seed and stem) extract pretreatments in Groups 3, 4, 5, and 6, most especially, the stem extract pretreatment in Groups 5 and 6, improved better the hepatic histoarchitecture, the hepatocellular, and the oxidative stress injury markers in a dose-dependent manner. Conclusively, ethanol extractions of* Cuscuta australis* stem appear to protect the liver from acetaminophen intoxication better than the seed counterpart.

## 1. Introduction

Acetaminophen (APAP) is a commonly used antipyretic analgesic drug for curing fever, headache, and other pains and is readily available without prescription. However, an overuse of acetaminophen has the potential of precipitating hepatic damage in both humans and animals [1]. The liver plays significant role in the metabolism of toxic chemicals, certain drugs, and environmental pollutants by using cytochrome P450 pathway. The latter convert acetaminophen to a highly toxic metabolite, specifically* N*-acetyl-*p*-benzoquinamine (NAPQI) which under normal conditions is readily detoxified by conjugation with glutathione (GSH) [2, 3]. Sustained overuse of acetaminophen enhanced the buildup of NAPQI which covalently bind to cellular macro molecules (proteins and DNA) to produce protein adduct that culminates in acute hepatic necrosis [4–6]. Oxidative stress is usually occasioned by increased level of this highly reactive species, NAPQI, and may contribute to the APAP hepatotoxicity via lipid peroxidation, mitochondrial damage, and ATP depletion [7]. Therefore, striking balance between reactive species and antioxidant enzymes might be a critical mechanism for ameliorating damage by oxidative stress under APAP toxicity [8]. Natural remedies from traditional plants are seen as effective and safe alternative treatments for hepatotoxicity [9].

*Cuscuta australis* is an annual parasitic plant that wraps around other plants for nourishment and forms the major flora of the tropical East and West Africa, Japan, and Australia [10].* C. australis* is locally named “Omonigelegele” among the Southwestern inhabitant of Nigeria where the complete plant (seed and stem components) preparation holds a folkloric significance against various health challenges [11].* C. australis* seed has been demonstrated to contain both kaempferol and astragalin majorly while the stem contains a high level of kaempferol [12]. In a similar study, Yen et al. [13] reported the hepatoprotective and antioxidant effects of* Cuscuta chinensis* against acetaminophen-induced hepatotoxicity in rats. However, there is no scientific documentation on the ameliorative potential of* C. australis* on acetaminophen-induced hepatotoxicity; this study seeks to investigate the possibility.

## 2. Materials and Methods

### 2.1. Chemicals

Chemicals were purchased from Sigma-Aldrich Chemical Co. (St. Louis, Missouri, USA): nicotinamide adenine dinucleotide phosphate (NADP); glycylglycine; glucose-6-phosphate; l-*γ*-glutamyl-3-carboxyl-4-nitronilide; epinephrine; hydrogen peroxide; glutathione (GSH); 5,50-dithio-bis-2-nitrobenzoic acid; thiobarbituric acid; and 1-chloro-2,4-dinitrobenzene. Acetaminophen was from Panadol, GlaxoSmithKline Consumer Plc. Every other reagent used was of analytic grade and was obtained from the British Drug Houses (Poole, Dorset, UK).

### 2.2. Plant Material Collection and Authentication

*Cuscuta australis* plant (seed and stem) was harvested from flower leaves within Agogo Ide compound, Abeokuta, Nigeria, and authenticated in the Department of Botany, University of Ibadan, with voucher specimen number UIH-22351.

### 2.3. Plant Extract Preparation

Three hundred (300 g) grams of* Cuscuta australis* of each sample was dried at room temperature, blended into powder, and mixed with 300 mL of ethanol using a Soxhlet extractor. The extraction runs for 6 hours allowing the extracts to mix effectively with the solvent at 20°C. The resultant filtrate was concentrated using a rotary evaporator (Bibby Sterling, Germany) to give 18.34 g residue weight of seed and 15.92 g of stem, yields of 6.11% and 5.31% (w/w), respectively.

### 2.4. Animal Protocol

A total of thirty-six Wistar rats (12 weeks) obtained from the experimental Animal Unit of the Faculty of Veterinary Medicine, University of Ibadan, Nigeria, were used for this study. The rats weighed between 150 and 200 g and maintained in galvanized wire mesh cages, under hygienic conditions. All animals were fed with pelletised growers mash and water* ad libitum*. They were acclimatized for two weeks prior to the commencement of the experiment. All rats received humane care in accordance to the “guide for the care and use of lab animals” (National Academic Press, Washington DC, USA, 1996).

### 2.5. Experimental Design

The modified experimental procedure of Yen et al. [13] was adopted. Rats were randomly divided into six groups, each consisting of six rats.

 Group 1: served as normal control and orally received sterile water for seven days and then intraperitoneally injected with 10 mL/kg body weight isotonic 0.9% NaCl.
 Group 2: served as hepatoxicity control and orally given sterile water for seven days and then intraperitoneally intoxicated with 835 mg/kg body weight acetaminophen on the 8th day.
 Group 3: this group was treated orally with low dose (125 mg) of* C. australis* seed extract for seven days and then intoxicated with 835 mg/kg acetaminophen on the 8th day.
 Group 4: this group was orally given high dose (250 mg) of* C. australis* seed extract for seven days and then intoxicated with 835 mg/kg acetaminophen on the 8th day.
 Group 5: this group was orally administered low dose (125 mg) of* C. australis* stem extract for seven days and then intoxicated with 835 mg/kg on the 8th day.
 Group 6: this group was treated orally with high dose (250 mg) of* C. australis* stem extract for seven days and then intoxicated with 835 mg/kg acetaminophen on the 8th day. Acetaminophen was dissolved in 40% polyethylene glycerol 400 for administration.

After 24 hours of acetaminophen intoxication, the rats were sacrificed for the evaluation of biochemical and histological variations in the liver tissues.

### 2.6. Biochemical Assays

Aspartate aminotransferase (AST) and alanine aminotransferase (ALT) were estimated spectrophotometrically as described by Toro and Ackermann [14] and Duncan et al. [15]. Total bilirubin level was determined by diazo reaction [16, 17]. Six sections of liver and kidney per experimental group of rats were homogenized in 50 mM Tris-HCl buffer (pH 7.4) containing 1.15% potassium chloride. The homogenate was centrifuged at 10,000 g for 15 min at 4°C. The supernatant was collected for the estimation of superoxide dismutase (SOD) by the method described by Misra and Fridovich [18]. GSH was determined at 412 nm using the method described by Jollow et al. [19]. Glutathione peroxidase assay was determined according to the method of Rotruck [20]. Lipid peroxidation was quantified as MDA according to the method described by Farombi et al. [21] and expressed as *μ*m MDA/g tissue. Catalase activities were assessed by the method of Clairborne [22].

### 2.7. Histopathological Preparations of the Tissues

Ten percent formalin fixed liver tissues were routinely processed and stained with haematoxylin and eosin for light microscopy at ×100 and ×400 magnifications.

### 2.8. Statistical Analysis

The data was statistically analysed using one-way analysis of variance (ANOVA) and Turkey was used for multiple comparisons* post hoc*. The results were expressed as group mean ± standard error of mean, while the level of significance was *P* < 0.05.

## 3. Result

### 3.1. Histopathology

**Figure 1:**
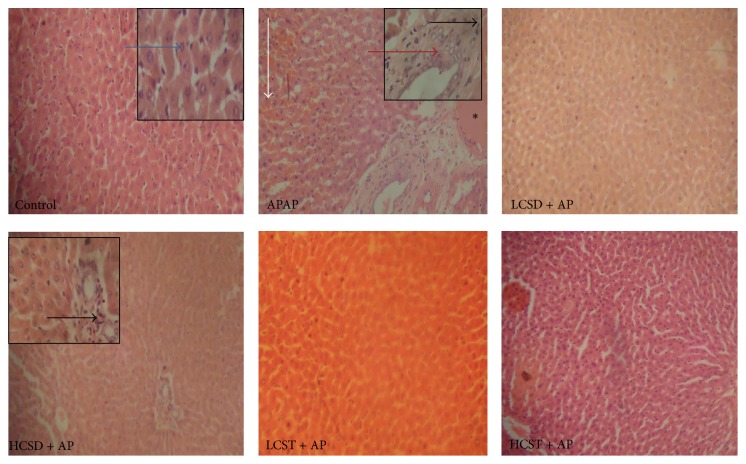
Photomicrographs of liver of acetaminophen-intoxicated rats and its amelioration by ethanolic extracts of* Cuscuta australis* seed and stem. Control: normal hepatic histoarchitecture (blue arrow); APAP/AP (acetaminophen): moderate portal congestion (asterisk) with severe periportal hepatic necrosis (red arrow), cellular infiltration (black arrow), and foci of hemorrhage in the hepatic parenchyma (white arrow); LCSD (low dose (125 mg) of* Cuscuta australis* seed) + AP: no visible lesion; HCSD (high dose (250 mg) of* Cuscuta australis* seed) + AP: periportal cellular infiltration (black arrow). LCST/HCST (low dose (125)/high dose (250 mg) of* Cuscuta australis* stem) + AP, respectively, showed normal hepatic histoarchitecture. Magnification: ×100; inset pictures at M ×400.

The liver of the APAP exclusively intoxicated rats showed moderate portal congestion with periportal hepatic necrosis, cellular infiltration, and foci of haemorrhage into the hepatic parenchyma. The severity of APAP damage was reduced in the liver of group that received 250 mg dose of* Cuscuta australis* seed pretreatment. However, normal cytoarchitecture was observed in the low dose of the seed and in both doses of* Cuscuta australis* stem, respectively, as shown in Figure 1.

### 3.2. Serological Parameters

Table 1 shows the effects of pretreatment with* C. australis* against acetaminophen-induced variations on serological parameters. The hepatic injury markers, serum ALT, AST, and bilirubin levels, significantly increased (*P* < 0.001) in APAP-induced Group B rats relative to control. Pretreatment with 125 or 250 mg/kg extracts of* C. australis* seed significantly (*P* < 0.05 and *P* < 0.01, resp.) reduced the elevations. Similarly, doses of stem extract pair significantly (*P* < 0.05 and *P* < 0.001, resp.) ameliorated the elevated values in dose-dependent manner compared to exclusively APAP-intoxicated Group B rats.

### 3.3. Biochemical Parameters

**Figure 2:**
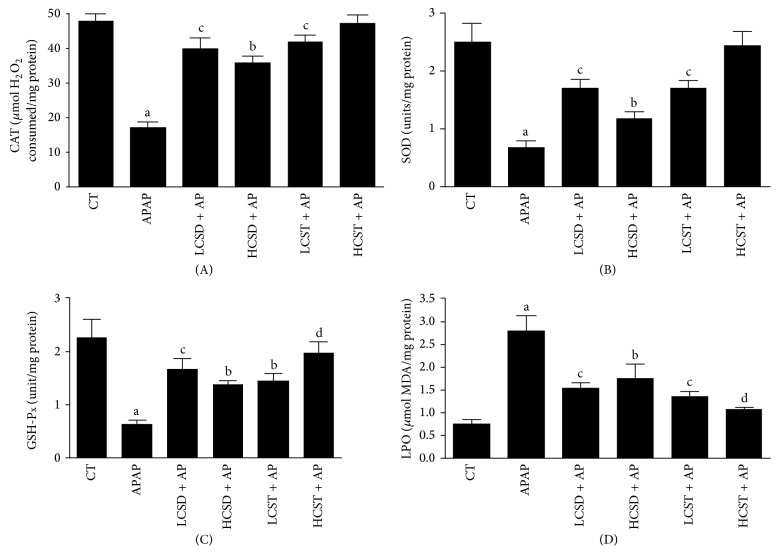
The effects of* Cuscuta australis *pretreatments on the antioxidant systems ((A) catalase (CAT), (B) superoxide dismutase (SOD), (C) glutathione peroxidase (GSH-P_X_), and (D) lipid peroxidation LPO) in hepatic tissues of acetaminophen-intoxicated rats. CT (control), APAP/AP (acetaminophen), LCSD/HCSD (low dose (125 mg)/high dose (250 mg) of* Cuscuta australis* seed), and LCST/HCST (low dose (125 mg)/high dose (250 mg) of* Cuscuta australis* stem). Values with different superscripts are significantly different; *P* values: a < 0.001: APAP compared with normal control; b < 0.05, c < 0.01, and d < 0.001: experimental groups compared with APAP.

The data on the effects of* C. australis* pretreatments against acetaminophen (APAP) induced antioxidant systems damage and lipid peroxidation in liver tissues (Figures 2(A)–2(D)) showed that APAP-intoxication significantly (*P* < 0.001) decreased the hepatic catalase (CAT), superoxide dismutase (SOD), and glutathione peroxidase (GSH-P_X_) enzymes of exclusively APAP-intoxicated group compared to control (Figures 2(A)–2(C)). However, levels of CAT, SOD, and GSH-P_X_ enzymes of rats given low (125 mg) and high (250 mg) doses of* C. australis* seed extracts significantly (*P* < 0.01 and *P* < 0.05, resp.) increased with strong efficacy at low dose when compared to the exclusively APAP-intoxicated group (Figures 2(A)–2(C)). The profile of CAT, SOD, and GSH-P_X_ enzymes of* C. australis* stem extracts was similar to its seed counterpart but a significantly (*P* < 0.01) dose-dependent response was observed in the stem extract pairs. The lipid peroxidation increased significantly (*P* < 0.001) in the APAP-intoxicated group relative to the control. The LPO level of the cotreated groups was strikingly ameliorated by varied doses of either extracts of* C. australis*. Although, cotreated group that received pretreatment with 400 mg dose of* C. australis* stem was insignificantly (*P* > 0.05) different from the control (Figure 2(D)).

## 4. Discussion

This study demonstrated that APAP-intoxication resulted in an overt hepatotoxicity as evidenced by serum elevations of ALT, AST, and bilirubins. Hepatotoxicity was also reflected in the liver as marked reduction in activities of GSH-P_X_, SOD, and catalase and significant elevation of lipid peroxides. The time point chosen in this work was based upon the known onset of the maximum hepatic injury induced by APAP-intoxication [13, 23]. Thus, the observed hepatotoxicity effects of APAP in rats were similar to the previously reported works [4, 6, 24].

The increased hepatic enzymes (ALT and AST) are biochemical markers of acute hepatocellular injuries. Their elevated serum levels reflect the extent of hepatocellular membrane damage and leakage [25]. In the same vein, changes in the serum levels of bilirubin points to the morphological integrity of the hepatocytes [15]. In this study, the observed elevated serum hepatic enzymes and bilirubin levels that follow APAP-intoxication further establish the widely reported effects of APAP-intoxication on liver biochemical markers [6, 9, 13, 26]. In addition, histopathological lesions of periportal hepatic necrosis and cellular infiltration and foci of hemorrhage into the hepatic parenchyma further strengthened the typical hepatic biochemical markers in APAP-intoxication. These findings corroborate the documentation of Yen et al. [13] and Sabeena and Ajay [23]. Pretreatment with varied doses (125 and 250 mg) of either extracts of* C. australis* protects the hepatocytes morphological integrities against APAP-intoxication as shown by drastic reduction of the hepatic enzymes (ALT and AST), bilirubin levels, and improved histoarchitecture. Comparatively, the crude extract of* Cuscuta australis* stem offers a better dose-dependent response to this morphological integrity protection compared to its seed counterparts.

These observations are suggested to be connected to the flavonoids (antioxidative agents) contents in the seed (astragalin and kaempferol) and stem (majorly kaempferol) of* C. australis* as earlier reported by Ye et al. [12]. However, further studies are needed to estimate the different flavones contents of our stem and seed preparations as their levels in Cuscuta species have been reported to be influenced by the host plants [12].

Oxidative stress is a major mechanism in the development of APAP-induced hepatotoxicity [27, 28]. It is usually occasioned by increased level of highly reactive* N*-acetyl-*p*- benzoquinamine (NAPQI) in cytochrome P450 which eventually weakened antioxidant defense system [3]. Preventive antioxidant enzymes such as superoxide dismutase (SOD), catalase (CAT), and glutathione peroxidase (GP_X_) are involved in direct elimination of reactive oxygen species, namely, superoxide radical and hydrogen peroxide that emanated from the disruptive effect of NAPQI [29]. SOD represents the first line of defense that assists in mopping up singlet oxygen and splitting superoxide radicals to H_2_O_2_. The elimination of the latter is usually carried out by hepatic catalase which progresses the course of scavenging the hydrogen peroxide produced while glutathione peroxidase through its catalytic action on lipid peroxides prevents the formation of lipid peroxidation reaction [30]. The result on SOD, CAT, and GP_X_ antioxidant enzymes as reflected by decreased levels of these enzymes in exclusively APAP-intoxicated rats further established the toxic potential of APAP. These findings agree with popular reports of antioxidant enzymes depletion in APAP-intoxication [9, 13, 24]. These depletions disrupted the hepatic defense systems and thereby favor excessive accumulation of H_2_O_2_ which eventually weakened the system. Similarly, hepatic lipid peroxidation (LPO) level is associated with the production of superoxide radical. Therefore, the increased level of LPO in this study suggested that APAP-intoxication stimulates free radical-generating ability of the liver tissues of APAP-intoxicated rats.

Interestingly,* C. australis* pretreatments, most especially the stem extract, remarkably ameliorated the disrupted antioxidant defense systems in dose-dependent manner by improving the levels of APAP-intoxicated depleted antioxidant enzymes (CAT, SOD, and GP_X_) and quantity of LPO generated in the liver. These observed ameliorations are suggested to be linked to the phytoantioxidants constituents (astragalin and kaempferol) of* C. australis* and particularly the high concentration of kaempferol in the stem of* C. australis.* The hepatoprotective effects of* C. australis* observed in this study are similar to the previous report on* Cuscuta chinensis* [13] in a rat model of acetaminophen-induced hepatotoxicity. This is the first report on hepatoprotective effects of the stem and seed of* C. australis* and provides a new insight into the traditional use of this parasitic vine as a supplement for the management of liver diseases.

## Figures and Tables

**Table 1:** Effect of *Cuscuta australis* extracts on liver function markers in acetaminophen-intoxicated rats.

Treatment	AST	ALT	TBIL
(A) CONTROL	19.00 ± 2.86	10.80 ± 0.83	0.33 ± 0.04
(B) APAP (865 mg)	93.50 ± 11.66^a^	50.67 ± 8.68^a^	1.18 ± 0.14^a^
(C) LCSD + AP	36.70 ± 3.03^c^	19.00 ± 2.35^c^	0.54 ± 0.01^b^
(D) HCSD + AP	54.87 ± 5.19^b^	26.33 ± 3.24^b^	0.72 ± 0.07^b^
(E) LCST + AP	45.83 ± 3.91^b^	14.40 ± 1.20^c^	0.62 ± 0.09^b^
(F) HCSD + AP	23.00 ± 2.28^d^	10.67 ± 0.99	0.43 ± 0.04^c^

AST: aspartate aminotransferase; ALT: alanine aminotransferase; TBIL: Total Bilirubin; LCSD/HCSD: (Low dose (125 mg)/High dose (250 mg) of *Cuscuta australis* seed); LCST/HCST: (Low dose (125 mg)/High dose (250 mg) of *Cuscuta australis* stem). Values with different superscripts are significantly different; *P-*values: ^a^ *P* < 0.001: APAP compared with normal control; ^b^ *P* < 0.05, ^c^ *P* < 0.01, ^d^ *P* < 0.001: experimental groups compared with APAP.
